# Supplementary material for: Acute Toxicity of the Hydroethanolic Extract of the Flowers of Acmella oleracea L. in Zebrafish (Danio rerio): Behavioral and Histopathological Studies
Source: Pharmaceuticals (Basel). 2019 Nov 27;12(4):173. doi: 10.3390/ph12040173 (PMC6958448; doi:10.3390/ph12040173)
Supplement: Supplementary file 1 [file pharmaceuticals-12-00173-s001.pdf]

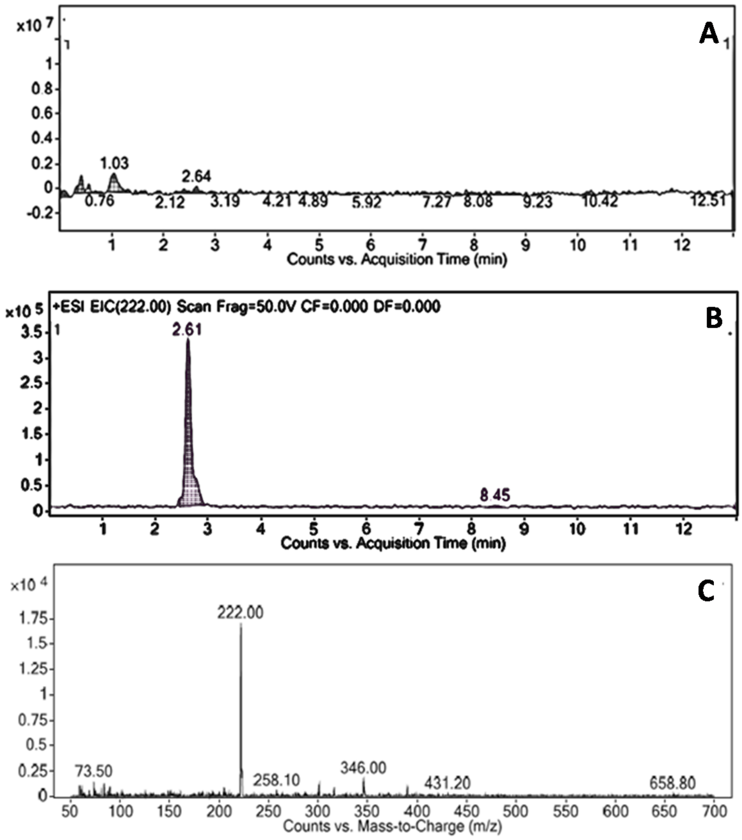

Figure supplementary 1. UHPLC-DAD-ESI-MS analysis: Total ions chromatogram (**A**); Chromatogram Ion extracted (B) and the mass spectrum obtained in Scan mode (**C**) of the hydroethanolic fraction of EHFAo. Souza et al. [29].
